# RNA-seq research landscape in Africa: systematic review reveals disparities and opportunities

**DOI:** 10.1186/s40001-023-01206-3

**Published:** 2023-07-22

**Authors:** Albert Doughan, Wisdom Adingo, Samson Pandam Salifu

**Affiliations:** 1 Department of Biochemistry and Biotechnology, Kwame Nkrumah University of Science and Technology (KNUST), Kumasi, Ghana; 2 Kumasi Centre for Collaborative Research in Tropical Medicine (KCCR), Kumasi, Ghana

## Abstract

RNA sequencing has emerged as the standard method for transcriptome profiling of several human diseases. We performed a systematic review detailing the state of RNA-seq analyses in Africa from its inception till February 2022. Our goal was to provide an update on the state of RNA-seq analyses in Africa, including research gaps, funding information, participants information, authorship and collaborations. Following the PRISMA guidelines, we performed an exhaustive literature search for RNA-seq studies conducted in Africa, using PubMed, Scopus and Academic Search Complete (EBSCOhost). The output was exported to Endnote X9 for analyses. The initial literature search yielded 10,369 articles spread across PubMed (4916), Scopus (4847) and EBSCOhost (580). By applying our exclusion criteria, 28 full-text articles remained and were thoroughly analyzed. Overall, 17 human diseases were studied, including cancers (10/28), infectious disease (4/28), parasitic disease (4/28), autoimmune disorders (2/28) and neglected tropical diseases (2/28). Majority of the articles were published in PLoS Pathogens, BioMed Central and Nature. The National Institutes of Health (42.4%), the Bill & Melinda Gates Foundation (7.5%) and the Wellcome Trust (7.5%) were the top funders of the research studies. Eleven African countries contributed to the participant group, with 57% located in Eastern Africa, 23.1% from Western and 16.7% from Southern Africa. The extremely low number of RNA-seq research studies in Africa is worrying and calls for an immediate investment in research by the African governments. The funding agencies and institutional review boards should also ensure that African collaborators are treated equitably in the course of the research projects.

**Supplementary Information:**

The online version contains supplementary material available at 10.1186/s40001-023-01206-3.

## Introduction

Gene expression profiling (GEP) has been widely employed in biological research, leading to substantial breakthroughs in our understanding of the molecular mechanisms behind complex human diseases such as cancer, autoimmune and heart diseases, and metabolic disorders [1]. To gain insight into a cell's transcriptome, RNA sequencing (RNA-seq) method is commonly employed. In comparison with previous gene expression measures such as microarray analyses, RNA-seq approaches can discover new transcripts, identify alternatively spliced genes, detect allele-specific expression and quantify gene expression [2]. This makes it a versatile technology for gene expression profiling in various conditions. RNA-seq was initially introduced in 2008 [3, 4] and has grown in popularity over the last decade. This could largely be attributed to the dramatic reduction in the cost of sequencing [5] and the availability of sequencing platforms in several institutions worldwide [6].

RNA-seq studies have made significant contributions to several fields, particularly cancer research [7], including differential gene expression analysis and cancer biomarkers identification [8], cancer drug resistance [9], cancer heterogeneity and evolution [10] and cancer microenvironment and immunotherapy [11]. For RNA-seq analyses to effectively contribute to biomarker identification and precision medicine development, it is incumbent on scientists to consider the different world populations. Gene expression during a diseased state varies considerably from one geographical location, diet, climate, and even gender. As a result, profiling gene expression in as many geographical locations as possible is critical.

The gene expression profiles of western populations have been thoroughly studied; however, data on the expression profiles of African patients are limited. Due to context-specific characteristics, RNA-seq analyses that improve our understanding of major diseases must be replicated in Africa. Additionally, environmental variables such as living conditions, diet, pollution, climate and genetic heterogeneity that may influence gene expression are more pronounced in Africa [12]. As a result of these differences, it may not be prudent to generalize results from RNA-seq studies conducted in only one population.

Currently, to the best of our knowledge, no study has summarized RNA-seq studies performed within Africa in order to delineate potential researchable areas, gaps in knowledge, countries at the forefront of RNA-seq and those lagging in Africa. It is an undeniable fact that Africa is burdened with diseases such as malaria, neglected tropical diseases, tuberculosis, trypanosomiasis, and onchocerciasis, different from those of the developed world. This accentuates the need for health-related research to focus on these diseases.

In this study, we performed a systematic review of RNA-seq analyses that have been conducted on African patients with various human diseases. Our goals were to (1) summarize studies that implemented RNA-seq studies on African patients; (2) identify potential research gaps and future research options in RNA-seq analyses in Africa; (3) identify countries that are championing RNA-seq in Africa; (4) assess the contribution of African-based researchers; (5) ascertain whether the target diseases are relevant to Africans; (6) provide information on funding sources and the number of papers published by African-based scientists on RNA-seq analyses.

## Materials and methods

### Data sources and study selection

In strict adherence to the Preferred Reporting Items for Systematic Reviews and Meta-analyses (PRISMA) guidelines [13], two of the authors conducted a comprehensive literature search. We searched for published articles in three authoritative scientific databases: PubMed, Scopus and Academic Search Complete via EBSCOhost. Hand searches using Google and Microsoft Bing search engines were also undertaken. The search terms were broadly grouped based on the type of next-generation sequencing (NGS) technology, the relevant countries, years of publication and the type of species being studied. The complete list of the search terms for each database can be found in Supplementary file 1.

### Inclusion and exclusion criteria

The search results were considered eligible provided that:

- the studies employed bulk RNA-seq technology,
- the year of publication of the article was 2008 onwards,
- the study participants originated from Africa,
- the study was published in English language,
- the study was conducted on humans.

Articles with the following characteristics were excluded from the analyses:

- studies that employed gene quantification methods such as microarray,
- studies that performed single-cell RNA-seq,
- studies that analyzed publicly available RNA-seq data,
- studies where participants originated from countries other than an African country,
- studies that used animal models,
- studies published in languages other than English,
- review articles, letters to the editor, commentary, opinions, case studies and editorials.

### Quality assessment

**Fig. 1:**
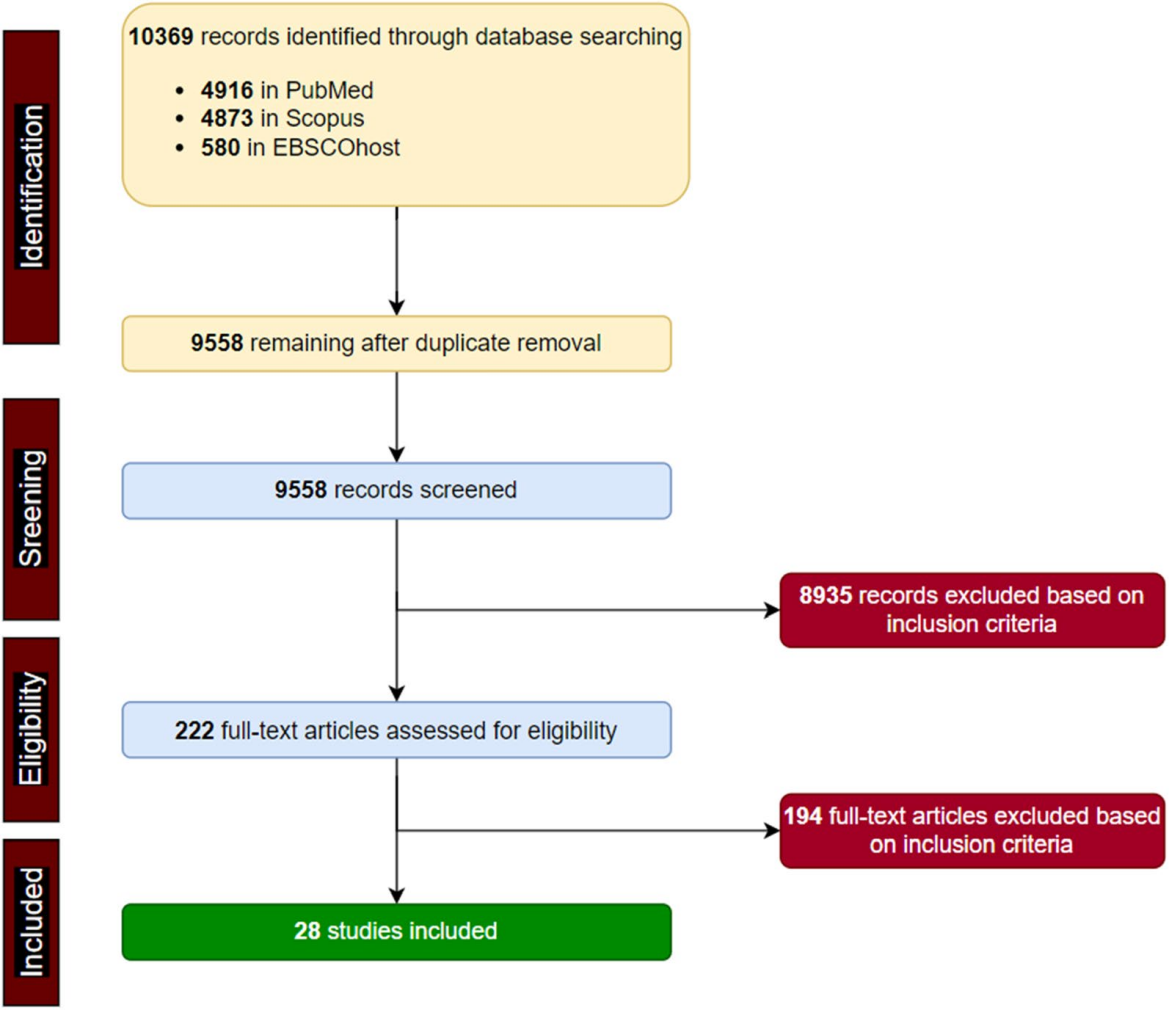
Flowchart of literature search for articles that employed RNA-seq analyses in African populations

Two reviewers conducted all the steps in (Fig. 1). The citations of all the search results were imported into Endnote X9. All duplicated articles were removed. Papers whose titles and abstracts were revealed to be irrelevant to the subject and for the purpose of the study were removed. All articles that failed the inclusion criteria were also removed. Results from both reviewers were compared until a consensus was reached. All authors addressed discrepancies in the results.

### Data extraction and synthesis

We extracted the following information from the relevant full-text articles obtained by the two reviewers; (a) the demographics of the study participants; (b) the author affiliations; (c) the disease type being studied; (d) the funding sources; (e) the journals in which the paper was published and; (f) the author’s information. By assessing the disease type being studied, our goal was to establish the extent to which the studies focused on diseases relevant to Africa, as defined by the Africa Centers for Disease Control and Prevention [14]. These include neglected tropical diseases, malaria, anthrax, tuberculosis, pneumonia, HIV/AIDS, Lassa fever and meningococcal meningitis. Regarding the funding sources, we focused on organizations that directly funded the project instead of scholarships awarded to one or more of the authors. We only reported on participants and researchers based in Africa in international projects.

## Results

### Literature search output

Following the PRISMA guidelines, an initial literature search yielded 10,369 articles consisting of PubMed (4916), Scopus (4847) and EBSCOhost (580). No article was found by hand searching using the two search engines (Google and Microsoft Bing). After duplicate removal, 9958 records remained and based on the set inclusion criteria, 8935 articles were removed. The full texts of 222 articles were assessed for eligibility, and 28 studies were finally arrived at and included in the study (Fig. 1).

### Journal information and diseases studied

**Fig. 2:**
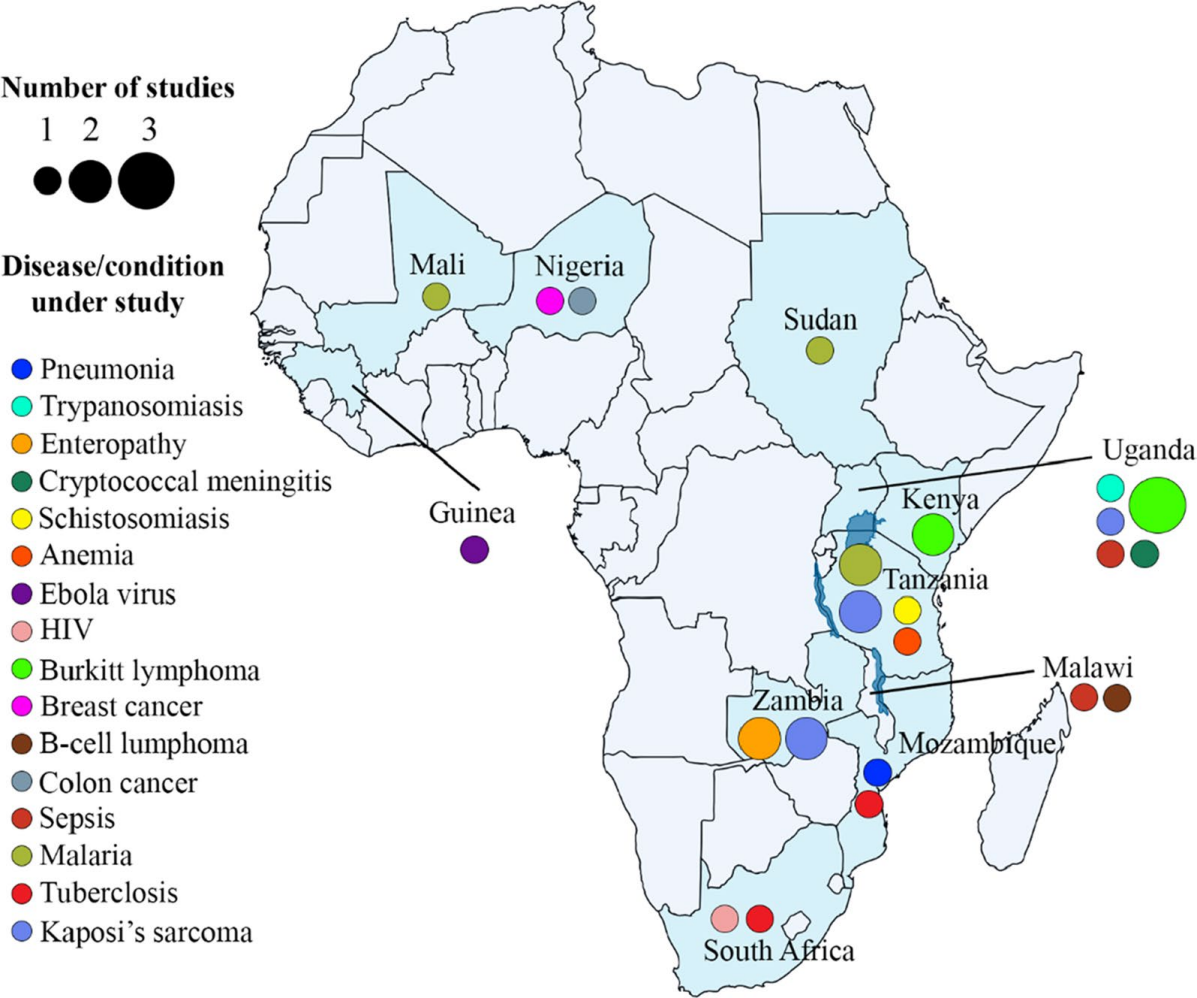
An African map showing the distribution of studies and the various diseases across eleven countries. The colored circles represent the disease type under study, whereas the size of the circles represent the number of studies conducted within a country

The 28 articles included in this study employed RNA-seq analyses to study the gene expression profiles of African patients. The authors adhered to the conventional RNA-seq study design (case–control), where two groups of individuals were studied; one group had the disease of interest, and the other group did not. Overall, 17 different diseases were studied, including cancers (10/28), infectious disease (4/28), parasitic disease (4/28), autoimmune disorders (2/28), neglected tropical diseases (2/28) and others (6/28) (Table 1). Burkitt lymphoma was the most studied of all the cancers (5/10) (Fig. 2). Majority of the studies employed DeSeq2 (11/28) and EdgeR (5/28) for the differential gene expression analysis with utilizing various statistically methods to unraveled the genes that are differentially expressed. Table 2 contains information about the differentially expressed genes specific to African population that were identified in each of the 28 studies, their p-values / false discovery rate (FDR) cut off, as well as the downstream analyses performed.

**Table 1:** Study characteristics, including body sites, sample types, diseases, funding and data availability

References	Participant’s country	Year	Authors’ countries	Body site	Sample type	Disease	Funding body	Data available?	Accession number	Participants number
Abate, Ambrosio [34]	Uganda	2015	Uganda, Kenya, Italy, United Kingdom, USA	Jaw, abdomen, neck	Biopsies	Burkitt lymphoma	Italian Association for Cancer Research, Programma Strategico, Steward Foundation, NIH	Yes	PRJNA292327	20
Ansari-Pour, Zheng [23]	Nigeria	2021	Nigeria, U.K., USA, Singapore, Spain	Breast	Biopsies	Breast cancer	NIH, Susan G. Komen for the Cure, Breast Cancer Research Foundation	Yes	phs001687.v1.p1	97
Chama, Amadi [35]	Zambia	2019	Zambia, USA, U.K	Intestine	Biopsies	Enteropathy	Bill & Melinda Gates Foundation	No	N/A	27
Cummings, Bakamutumaho [25]	Uganda	2022	Uganda, USA	Blood	Blood	Sepsis	National Center for Advancing Translational Sciences, National Institute of Allergy and Infectious Diseases, NIH	Yes	PRJNA794277	128
Duffy, Du [36]	Tanzania	2021	Tanzania, USA, Switzerland	Blood	Blood	Malaria	National Institute of Allergy and Infectious Diseases, Bill and Melinda Gates Foundation	N/A	N/A	33
Dupnik, Reust [37]	Tanzania	2018	Tanzania, USA, Netherlands	Blood	Blood	Schistosomiasis	NIH	N/A	N/A	33
Estévez, Anibarro [38]	Mozambique	2020	Mozambique, Spain, UK	Blood	Blood	Tuberculosis	EU Horizon2020 Eliciting Mucosal Immunity in Tuberculosis, Xunta de Galicia Grupo DE referencia Competitiva 2016, Spanish Ministry of Education	Yes	E-MTAB-7830	N/A
Fedoriw, Selitsky [39]	Malawi	2020	Malawi, USA	N/A	Biopsies	Diffuse large B-cell lymphoma	NIH	N/A	N/A	32
Fisher, Smith [40]	South Africa	2015	South Africa, USA	Blood	Blood	HIV	University of California, San Diego, Center for AIDS Research sub award, Polio Research Foundation, National Health Laboratory Research Trust (South Africa), NIH	N/A	N/A	15
Hatem, Hjort [41]	Tanzania	2022	Tanzania, Denmark, Finland	Blood	Blood	Anemia	Danish Council for Strategic Research	Upon request	N/A	50
Mahady, Kanabar [42]	Nigeria	2021	Nigeria, USA	Blood	Colon	Colon cancer	First Analysis Institute of Integrative Studies, Regenstein Foundation	N/A	N/A	
Kaymaz, Oduor [29]	Kenya	2017	Kenya, USA	Abdomen, Jaw	Fine needle aspirates	Burkitt lymphoma	NIH, The Thrasher Research Fund, Turkish Ministry of National Education	N/A	N/A	28
Kelly, Amadi [43]	Zambia	2021	Zambia, USA	Mucous	Biopsies	Environmental enteropathy	N/A	Yes	GEO 162630	27
Lai, Cortes [44]	South Africa	2021	South Africa, UK	Throat	Sputum	Tuberculosis	UK Medical Research Council, Cancer Research UK, Wellcome Trust	Yes	PRJEB10919	26
Popescu, Tembo [45]	Malawi	2020	Malawi, Canada, USA, Uganda	Blood	Blood	Sepsis	Grand Challenges Canada	N/A	N/A	18
Lidenge, Kossenkov [46]	Tanzania, Zambia	2020	Tanzania, Zambia, USA	Skin	Biopsies	Kaposi's sarcoma	NIH	Yes	GSE147704	24
Liu, Speranza [47]	Guinea	2017	UK, US, Germany, France, Guinea, Belgium	Blood	Blood	Ebola virus disease	NIH	Yes	PRJNA352396	128
Lombardo, Coffey [48]	Uganda	2017	USA, Ghana, Uganda	Mandible	Biopsies	Burkitt lymphoma	NIH	Yes	SRP099346	19
Mulindwa, Matovu [49]	Uganda	2020	Uganda, Germany	Spinal cord	Blood, spinal fluid	Trypanosomiasis	Deutsche Forschunggemeinschaft Germany–Africa project, AAS-Wellcome trust grant	Yes	E-MTAB-5293, E-MTAB-5294	9
Panea, Love [50]	Kenya	2019	Kenya, USA, Tanzania, Poland, China, Germany, Canada	N/A	Biopsies	Burkitt lymphoma	N/A	Yes	EGAS00001003778	32
Wichers, Tonkin-Hill [51]	Sudan	2021	Germany, UK, Malawi, Australia, Denmark	Blood	Blood	Malaria	Deutsche Forschungsgemeinschaft, Danish Council for Independent Research, Deutsches Zentrum für Infektionsforschung TTU Malaria, National Health and Medical Research Council, Wellcome Trust	Yes	PRJNA679547	32
Vlasova-St Louis, Musubire [52]	Uganda	2021	Uganda, USA	Blood	Blood	Cryptococcal meningitis	NIH	Yes	GSE162914	68
Tso, Kossenkov [53]	Tanzania, Zambia	2018	Tanzania, Zambia, USA	N/A	Biopsies	Kaposi's sarcoma	NIH	Yes	GSE100684	N/A
Tran, Jones [54]	Mali	2016	Netherlands, Mali, USA, Qatar	Blood	Blood	Malaria	NIH	N/A	N/A	8
Silterra, Gillette [55]	Mozambique	2017	Mozambique, Spain, USA	Blood	Blood	Pneumonia Syndrome	Bill & Melinda Gates Foundation	N/A	N/A	68
Schmitz, Young [56]	Uganda	2012	Uganda, USA, The Netherlands, Germany, Canada, Spain, Norway, UK	N/A	Biopsies	Burkitt lymphoma	NIH	N/A	N/A	13
Rothen, Murie [57]	Tanzania	2018	Tanzania, Switzerland, USA	Blood	Blood	Malaria	NIH	Yes	GSE97158	10
Rose, Bruce [58]	Uganda	2018	Uganda, USA	N/A	Biopsies	Kaposi’s sarcoma	NIH	Yes	GSE116160	41

**Table 2:** Study characteristics including Journal names, sequencing platforms and authors home countries

References	Journal	Sequencing platform	Statistical method	Downstream analyses method	FDR/P_adj_ cutoff	Africa-specific DEGs	Corresponding author	First author	Last author
Abate, Ambrosio [34]	PLoS Pathogen	Illumina HiScan SQ	SAVI algorithm	Characterization tumor microbiome with Pandora	N/A	MYC, ID3, TCF3, DDX3X, CCND3, TP53	USA, Italy	USA	USA
Ansari-Pour, Zheng [23]	Nature Communication	Illumina HiSeq2000	DESeq2	Somatic variant calling with Platypus	< 0.05	SYPL1, ZNF217, LAMB3, TP53, P1K3CA, GATA3, KMT2C	UK, USA	UK	USA
Chama, Amadi [35]	EBioMedicine	Illumina HiSeq2000	NOIseq	Pathway enrichment analyses with DAVID	N/A	DUOX2, DUOXA2, MUC1, SAA1, SAA2 SAA4, CXCL5	Zambia, UK	Zambia	Zambia, UK
Cummings, Bakamutumaho [25]	BMC Critical Care	Illumina HiSeq4000	DESeq2	Ingenuity pathway analysis	≤ 0.01	STAT3, PPAR, CD28, Nur77, OX40	USA	USA	USA
Duffy, Du [36]	BMC Malaria Journal	Illumina HiSeq2000	Limma	Gene-set enrichment analysis with fgsea R package	< 0.05	CKAP2L, DTL, EZH2, HJURP, NCAPH, NUSAP1, RRM2, SPAG5	USA	USA	USA
Dupnik, Reust [37]	Infection and Immunity	Illumina HiSeq4000	DESeq2	Ingenuity pathway analysis	≤ 0.05	CDKN2A, NDRG4, MIB2, NEURL, CTR9, HSF2, ELP2, WDR82, HDAC9	USA	USA	Tanzania, USA
Estévez, Anibarro [38]	Frontiers Immunology	Ion Torrent Proton Sequencer	DESeq2	Pathway enrichment analyses with Reactome, Machine Learning-Based Class-Prediction Analysis	< 0.05	C1QC, ADAMTS2, C1QB, METTL7B, DEFA3, PRR15, PRTNG	Spain	Spain	Spain
Fedoriw, Selitsky [39]	Nature Modern Pathology	Illumina HiSeq2000	ConsensusClusterPlus	Gene-set enrichment analysis with MSigDB,	< 0.1	N/A	USA	USA	USA, Malawi
Fisher, Smith [40]	Journal of Clinical Virology	Illumina MiSeq	N/A	Drug resistance mutational analyses	N/A	K103N, V106A, Y181C, K65R	South Africa	South Africa	South Africa
Hatem, Hjort [41]	The Journal of Clinical Endocrinology & Metabolism	Illumina Nextseq 500	edgeR	Gene-set enrichment analysis with Kolmogorov–Smirnov tests	< 0.05	LCORL, P2RX7, PIK3C2B, NUMBL, A2ML1, GPNMB, NUMBL, P2RX7, PIK3C2B, ADAMTSL5, ITGAD, NLRP1, PKD1L2	Sweden	Sweden	Sweden
Mahady, Kanabar [42]	Functional Foods in Health and Disease	Illumina NovaSeq 6000	edgeR	Gene ontology analyses with edgeR	≤ 0.05	PLGLB2, HCRT, TACR2, AXIN2, DRD1, ID2, RIMS2, CC2D2B, SP5, MISP	USA	USA	USA
Kaymaz, Oduor [29]	Molecular Cancer Research	Illumina HiSeq2000	DESeq2	Gene-set enrichment analysis with MSigDB, single nucleotide variation detection with GATK	< 0.1	CD19, CD20, CD10, CD79A/B, BCL6	USA	USA	USA
Kelly, Amadi [43]	EBioMedicine	Illumina HiSeq2000	NOIseq	Ingenuity pathway analysis	< 0.02	PRSS1, PRSS3, CPA2, CPA3, TMPRSS15, DPP4, GGT1	USA	UK, Zambia	USA
Lai, Cortes [44]	American Society for Microbiology Journals	Illumina HiSeq 2500	DESeq2	Ingenuity pathway analysis	< 0.1	CD8A, EOMES, LAG3, KLRC4-KLRK1, POU2AF1, FAIM3, KLRK1	South Africa, UK	UK	South Africa, UK
Popescu, Tembo [45]	Gates Open Research	Illumina HiSeq 2500	DESeq2	Ingenuity pathway analysis	< 0.1	PML, SOCS1, TICAM1, APOL1, GRINA, RMI2, ZBP1, IL27	Canada	Canada	Canada
Lidenge, Kossenkov [46]	PLoS Pathogens	Illumina Nextseq 500	DESeq2	Ingenuity pathway analysis	< 0.05	TLR8, MMP13, TMOD2. KCNQ3, ADAMTS1, CD8A, MCTP1, CEP19	USA	Tanzania, USA	USA
Liu, Speranza [47]	Genome Biology	Illumina HiSeq 2500	edgeR	Ingenuity pathway analysis	< 0.05	CXCL10, CCL2/MCP-1, CCL8/MCP2, CXCL11	UK	UK	UK
Lombardo, Coffey [48]	Blood Advances	Illumina HiSeq	DESeq2	Variant calling with GATK	N/A	ID3, TP53, SMARCA4, ZNF587, FOXO1	USA	USA	USA
Mulindwa, Matovu [49]	BMC Medical Genomics	Illumina NextSeq500	DESeq2	Pathway enrichment analyses with DAVID	< 0.1	C4BPA, FBN2, PROS1, TFPI, LAMC1, MYL9, RBPJ	Uganda	Uganda	Germany
Panea, Love [50]	Blood Advances	Illumina HiSeq 2500	Custom scripts	Copy number variation analysis with GATK4	N/A	IGLL5, BACH2, BTG2, BCL6, BCL7A, TCL1A, IRF8, CXCR4, ZFP36L1, and S1PR2	USA	USA	USA
Wichers, Tonkin-Hill [51]	Elife	Illumina HiSeq 2500	Limma	Functional enrichment analysis with gprofiler	< 0.05	MSP1, MSP2, MSP4, MSP10, EBA175, REX1, AMA1	Germany	Germany	Germany
Vlasova-St Louis, Musubire [52]	BMC Medical Genomics	Illumina HiSeq 2500	Cufflinks	Ingenuity pathway analysis	< 0.05	C1QA, C1QB, C1QC, CFD, CXCL1, CXCR1, ICAM1, IL6, IL8, IL11	USA	USA	USA
Tso, Kossenkov [53]	PLoS Pathogens	Illumina HiSeq 2500	DESeq2	Ingenuity pathway analysis	< 0.05	SEPT1, GPR182, MYLK2, COL10A1, NOS2, PROX1, CD177, MMP9, ADAM19, PALD1, ITGA9, TIE1	USA	USA	USA
Tran, Jones [54]	Scientific Reports	Illumina HiSeq2000	edgeR	Ingenuity pathway analysis	< 0.05	IL1RN, MAPK1, TRIM24, NKX2-3, TNF, IL1B, RELA, NFKB1A	USA	USA	USA
Silterra, Gillette [55]	The Journal of Infectious Diseases	Illumina HiSeq 2500	Support vector machine (SVM)	SVM modeling	N/A	N/A	USA	USA	USA
Schmitz, Young [56]	Nature	Illumina HiSeq2000	N/A	Mutational profiling, Gene copy number analysis	N/A	MYC, ID3, DDX3X, TCF3, SMARCA4, NCOR2, GNA13, MKI67, EXOSC6, WDR90	USA	USA	USA
Rothen, Murie [57]	PLoS One	Illumina HiSeq2000	edgeR	Hypergeometric gene testing with GeneOverlap R package	< 0.05	CSP, LSA-1, EXP-1, M143, M144, M147, M230, M237	Switzerland	Switzerland	USA
Rose, Bruce [58]	PLoS Pathogens	Illumina HiSeq 2500	N/A	Phylogenetic analysis	N/A	N/A	USA	USA	USA

NA means not reported

**Fig. 3:**
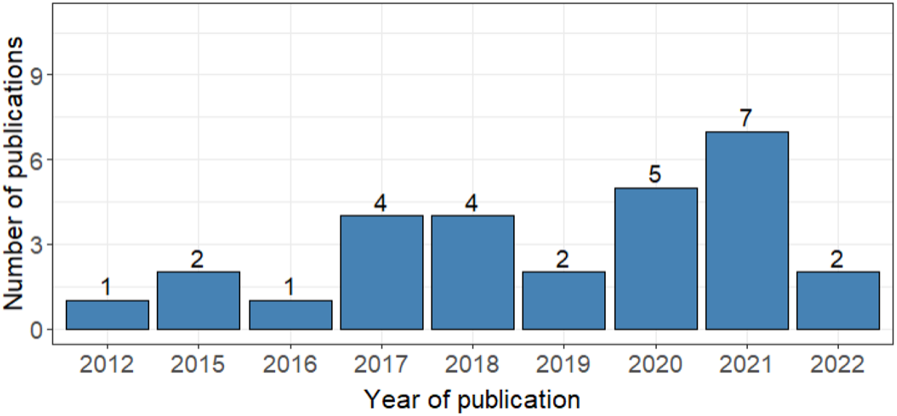
Number of published articles in a specific year

The articles were published in 23 different open-accessed journals, and only one is unavailable in PubMed Central. PLoS Pathogens, BioMed Central (BMC), Nature and Blood Advances contained the highest number of published articles with 17.9%, 14.3%, 14.3% and 7.1%, respectively (Table 2). The majority of the articles were published between 2017 and 2021 (22/28) (Fig. 3), and all the articles are freely available for download.

### Study participants’ information and distribution across Africa

**Fig. 4:**
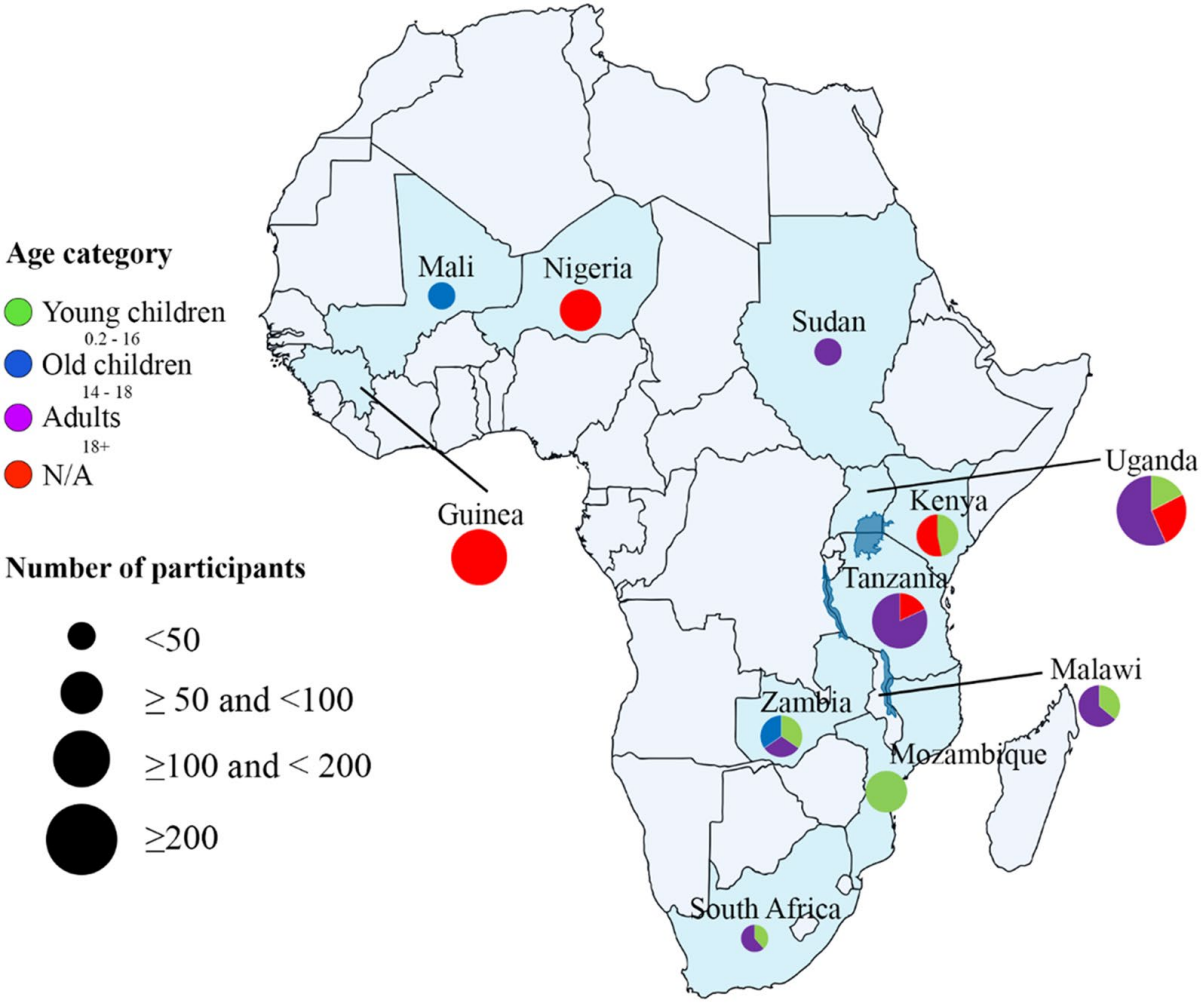
A map of Africa showing the number of study participants and their age groups across the various countries. The size of the circles depict the total number of study participants, while the different colors represent the different age groups

Overall, 986 participants of African descent were included in the studies. They were distributed across 11 countries in Western (233/1010), Eastern (576/1010), Southern (169/1010) and Northern (32/1010) Africa. Some studies recruited patients from two or more countries, which increased the total to 1010. The highest number of participants was recruited from Uganda (*n* = 298), Tanzania (150) and Guinea (128). Sudan and Mali had the least number of participants of 32 and 8, respectively. Sudan was the only Northern African country that recruited participants for the RNA-seq studies (Fig. 4). Approximately 61% of the studies had no information on the gender of the study participants. Of the remaining 39%, 62% were females, whereas 38% were males.

### Body site and sample type

**Fig. 5:**
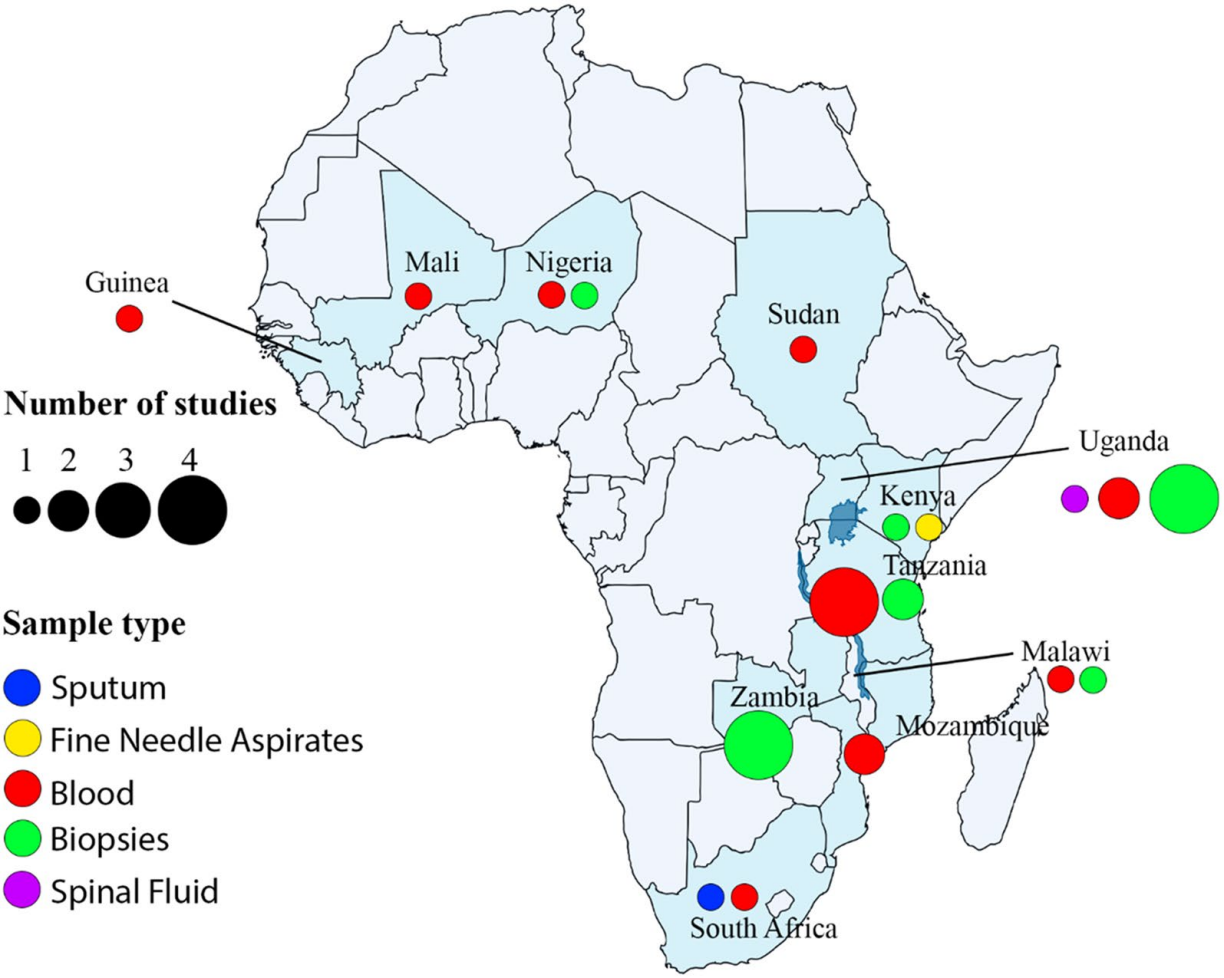
An African map showing the different sample types (colored circle) and the number of studies (circle sizes) that used a specific sample type

**Fig. 6:**
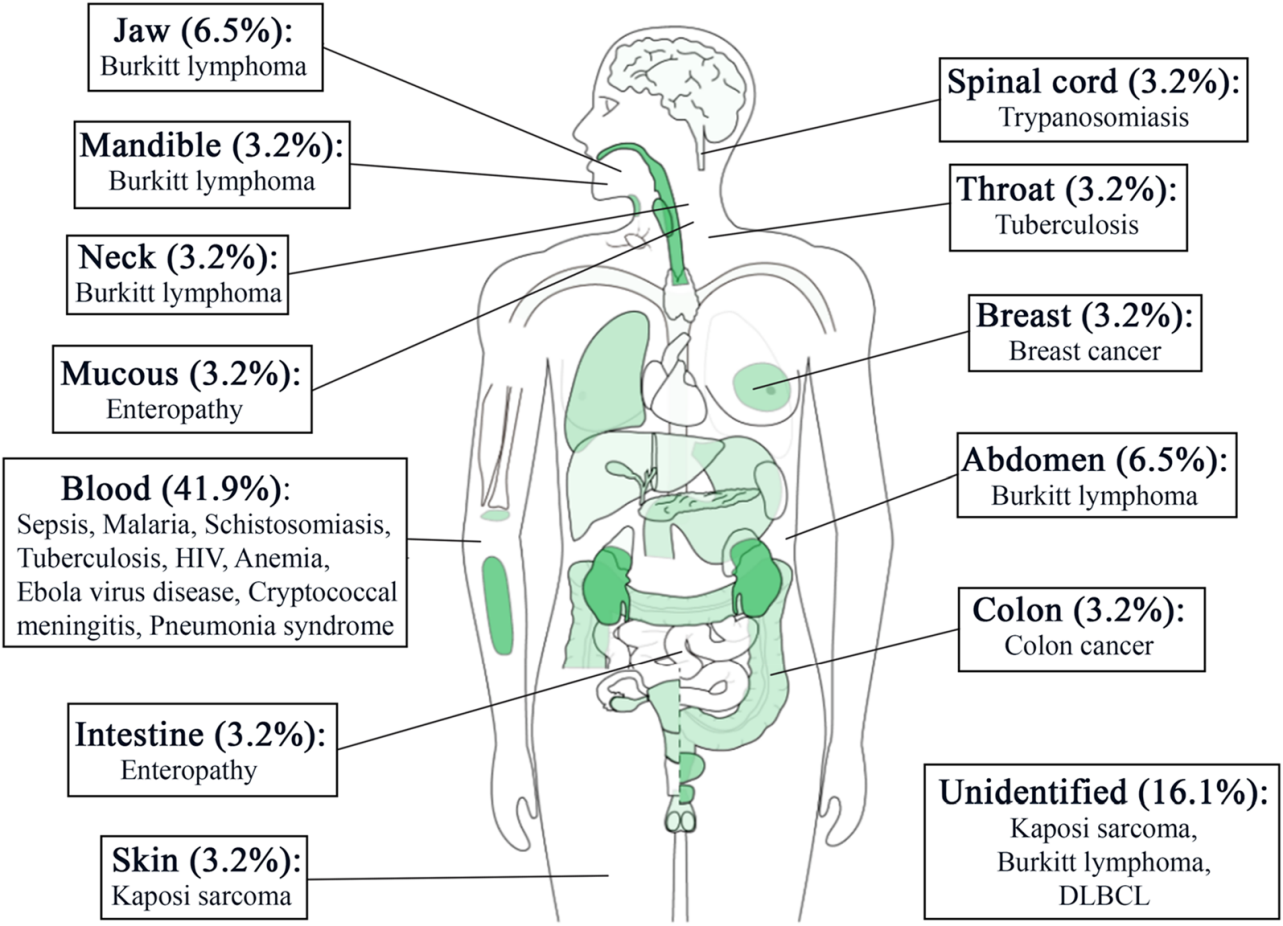
Graphical representation of the body sites from which the samples were taken for RNA extraction

Half of the studies (49.9%) collected blood from the participants (Figs. 4 and 5). Other sample types used included tissue biopsies (39.3%), fine needle aspirates (3.6%), spinal fluid (3.6%) and sputum (3.6%). The most widely studied body site was also blood (41.9%), followed by the jaw (6.5%) and abdomen (6.5%) (Figs. 4 and 5). The remaining body sites (neck, breast, mandible, mucous, intestine, skin, spinal cord, throat and the colon) each constituted about 3.2% (Fig. 6). Studies that did not provide information on the body sites where the samples were extracted comprised approximately 16.1%.

### Authorship information

**Fig. 7:**
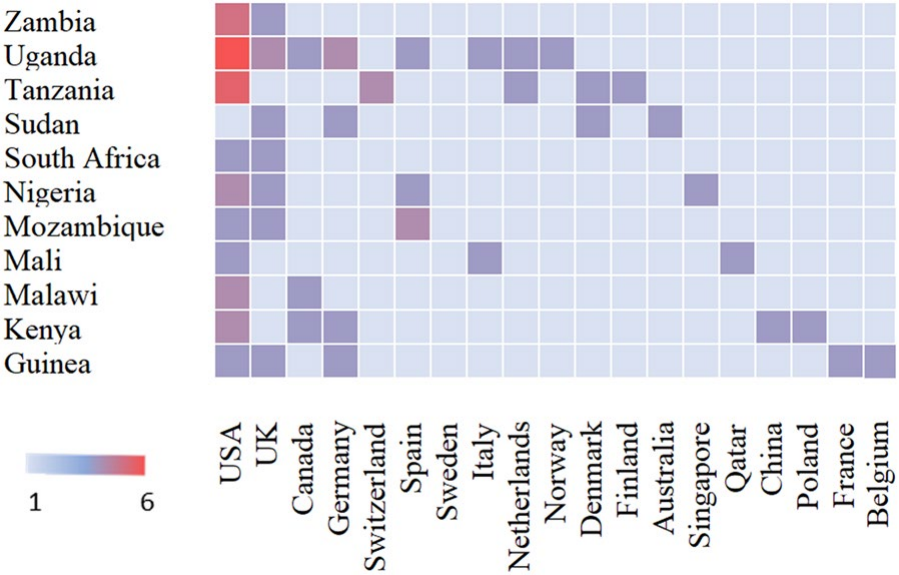
Graphical representation of the extent of collaborations between African-based and non-African researchers. The higher the extent of collaboration, the deeper the shade of red color

We explored information on the authors of the various articles with specific emphasis on the first, the last and the corresponding authors, as well as the extent of collaborations among African-based and non-African-based researchers (Fig. 7). Although first authors may not be authorities in their respective fields, they contribute most during manuscript writing and editing. Last authors and corresponding authors are seasoned researchers in their fields and usually serve supervisory and review roles. The corresponding authors of the 28 articles were unequally distributed across 11 countries from three continents (North America, 19; Europe, 9; and Africa, 4). Researchers in the USA accounted for over 67% of all the corresponding authors. This was followed by authors from the U.K. (12.5%) and South Africa (6.3%). Zambia, Spain, Sweden, Canada, Uganda, Germany, Switzerland and Italy had one corresponding author each. Regarding the first authorship, 53.3% were based in the USA, 13.3% were from the U.K., and 6.7% were from Zambia. The USA and the U.K. had the highest numbers of the last authors; 19 and 3, respectively. South Africa and Spain had two last authors each. In 75% of the articles, the corresponding authors doubled as the last authors (Table 2). The highest level of collaboration was observed between researchers from the USA and the following African countries; Uganda (6), Zambia (5), Tanzania (5), Nigeria (2), Malawi (2) and Kenya (2) (Fig. 7).

### Funding

**Fig. 8:**
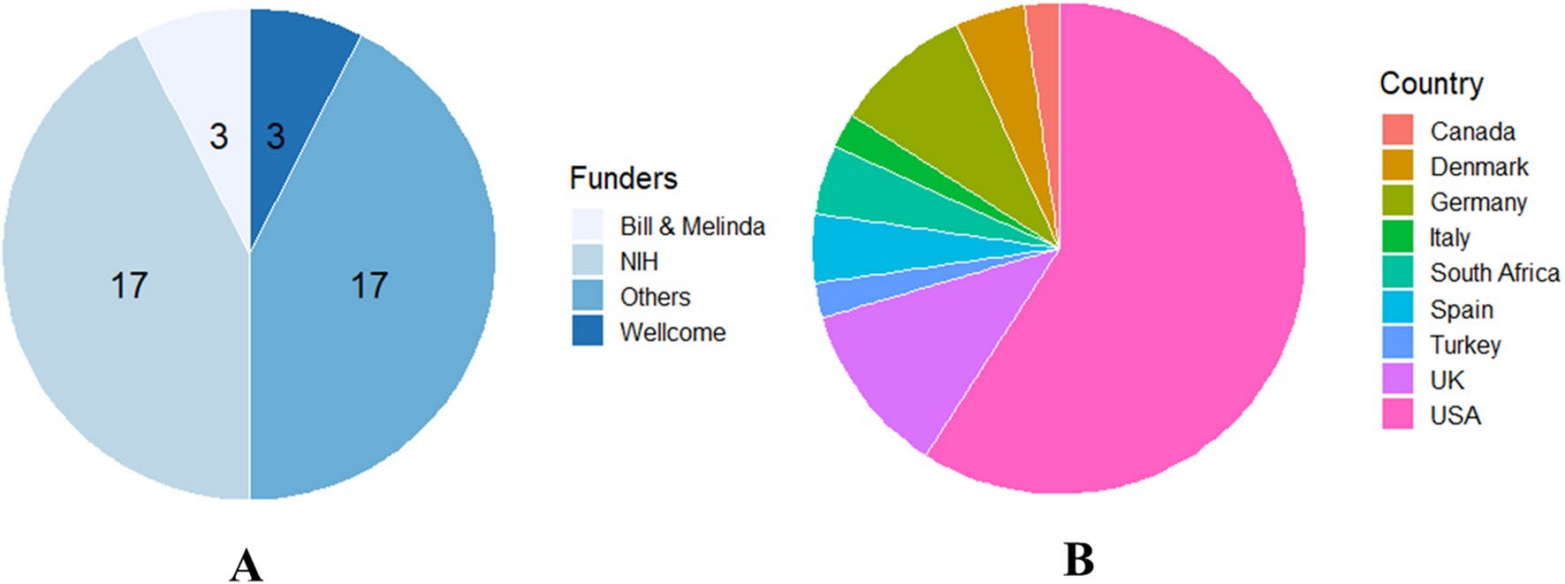
**A** Representation of the number of studies funded by international agencies and **B** the countries where the agencies are located

Table 1 and Fig. 8 summarize the organizations and agencies that awarded grants to the respective authors for the research projects in Africa. The grants were received from agencies within nine countries spread across three continents (North America, Europe and Africa). Funding agencies from the United States of America (USA), United Kingdom (U.K.) and Germany provided 59.1%, 11.4% and 9.1%, respectively, of the entire grants awarded to the authors. South Africa was the only African country whose government made grants available to support RNA-seq research projects, and they accounted for 4.5% of the total number of grants awarded.

Moreover, the funding bodies that contributed the most grants were the National Institutes of Health, NIH (42.4%), the Bill & Melinda Gates Foundation (7.5%) and the Wellcome Trust (7.5%). Five different agencies funded one study, 2 studies were supported by four agencies, 4 studies by three agencies, 4 studies by two agencies, 15 studies by one agency and 2 studies had no information on the funding sources. Grand Challenges Canada, the German Research Foundation, the Steward Foundation and the National Health Laboratory Research Trust (South Africa) were among the other funding bodies.

### Sequencing technologies and data availability

Regarding the availability of the RNA-seq data in public databases, 16/28 studies made the research data available with accession numbers, one was available upon reasonable request to the corresponding authors, one could not be made available due to identifying patient information, and 10 had no information on data availability. The 28 studies used NGS platforms from Illumina [27] and Thermo Fisher Scientific (1). Illumina HiSeq2000 was employed by 10/28 authors for sequencing, followed by Illumina HiSeq2500 (9/28), Illumina Nextseq500 (3) and Illumina HiSeq4000 (2). One study used Thermo Fisher’s Ion Torrent Proton sequencer (Table 2).

## Discussion

In the present systematic review, we aimed at surveying research projects within Africa that employed RNA-seq to analyze the gene expression profiles of African patients with various human diseases. Undertaking such analyses revealed the specific diseases that are widely studied and those that have been neglected. It also provided comprehensive information on the funding sources, countries with authors in the leading role, participants’ demographics, authors' affiliations and collaborations.

From 2008, when the first results of RNA-seq were published, till February 2022, 28 RNA-seq studies have been conducted where Africans formed part of the study participants. Considering the high prevalence of infectious and other parasitic diseases in Africa, we found the total number of studies to be quite disappointing and further accentuates the underrepresentation of Africans at the genomics level. Since its discovery, only eleven out of the 54 African countries have employed RNA-seq analysis to studies diseases affecting them. Interestingly, most of these studies were performed in Eastern Africa patients, while Northern Africa had a single study. This observation could be attributed to the fact that the studied diseases were not common in Northern Africa compared to the Western and Eastern parts of Africa. For instance, Burkitt lymphoma was the most widely studied disease, and it is highly prevalent in malaria-endemic regions such as Eastern and Western Africa [15]. It is possible that competent researchers in Northern Africa may prioritize other diseases using other sequencing technologies such as whole-exome or whole-genome sequencing.

Over 80% of the studies recruited less than 50 participants. Although six samples (participants) are sufficient to conclude RNA-seq studies [16], increasing the number of samples greatly increases the statistical power and culminates in concrete conclusions [17]. The inability of the researchers to include many participants may be due to financial constraints, including sequencing costs, consumables and wet lab analyses. One method that could have been employed to minimize the sequencing cost was multiplexing [18, 19], allowing simultaneous sequencing of multiple samples.

All the articles were published in either open-accessed or subscription-based journals with 27/28 were freely available through PubMed. The remaining article was also freely available for download on the Journal's website. The free availability of the articles could be due to the open-access revolution and the scientific community’s advocacy for open-access publication [20]. Most African universities and other academic institutions struggle to subscribe to first-class Journals due to the high cost involved. Open access publication will ensure that studies conducted in Africa will be freely available to students, policymakers, government officials and the entire scientific community. It also increases the visibility of African researchers on the global stage, which promotes collaborative research activities. As a result of the expensive nature of RNA-seq studies, these collaborations could acquire grants that can be used to study diseases that terrorize the African continent.

Studies that focused on the gene expression profiles of patients with cancer constituted 36% used RNA-seq. This was unexpected, as the majority of diseases that plague the African continent are parasitic diseases, infectious diseases and neglected tropical diseases, as stipulated by the African CDC [14]. Many of the funding agencies that underwrite the projects come with their already-defined objectives and diseases that can be studied. This makes it difficult for researchers based in Africa to focus on more common diseases in Africa. The various African governments and philanthropists can mitigate this by providing financial resources, which will be used to exclusively study some of these diseases that are prevalent in Africa, such as Malaria, Trypanosomiasis and other neglected tropical diseases. Although some funding agencies abroad allocate funds to study these diseases, they come with terms and conditions that may limit the nature of analyses to be performed.

The U.S. NIH made the most contribution (42%) in funding for the studies. The BMGF and Wellcome Trust also contributed some level of funding. Surprisingly, the South African government was the only African government that partially funded one of the 28 RNA-seq studies through the National Health Laboratory Research Trust. This observation is consistent with findings from Allali, Abotsi [21], who review the state of microbiome research in Africa. African governments and other funding bodies based in Africa need to understand the importance of research and its contribution to growth and development. Researchers based in Africa continue to rely on foreign grants to conduct research are less likely to enjoy the flexibility of studying diseases that threaten public health in Africa, such as malaria, neglected tropical diseases, cholera, Chikungunya, Dengue Fever, Ebola Virus Disease, Lassa Fever and typhoid fever. Additionally, these researchers may not receive the necessary recognition deemed them. This is evident in our study, where over 50% of the first, last and corresponding authors were based in the USA. On the other hand, only 12.5%, 16.7%, and 15.6% of the corresponding, the first and the last authors were based in Africa. If the African government invests in research by providing grants, African scientists can initiate and lead projects involving public health emergency diseases.

The sequencing platform used for most of the studies was Illumina. This was distributed among Hiseq2000, Hiseq2500 and Hiseq4000. The Illumina Hiseq system was launched in 2010 [22] and has since been the sequencing platform of choice for RNA-seq [23–26]. This could be attributed to the following; Illumina Hiseq has a multiplexing system, enabling thousands of different samples to be sequenced simultaneously. It also has an average error rate of 2% and a storage space of 3 T.B. (Hiseq2000). The qualities of the Illumina platform make it ideal for researchers in resource-constrained environments such as Africa.

More than 50% of the studies provided accession numbers to the RNA-seq data deposited in public repositories. To promote reproducibility, data deposition in public databases by authors has been championed by both the funding bodies [27] and Journals [28]. The data could also be used as secondary data for students and researchers, mainly those based in resource-constrained institutions such as Africa. Other research projects can pool several of these data, add to their generated data, and analyze, as seen in Kaymaz’s study on Burkitt lymphoma [29].

The 28 studies included in this review recruited 986 participants from Africa. Uganda and Tanzania had the highest number of participants, 298 and 150, respectively. Three reasons could account for this observation; the authors based in these African countries had collaborators in foreign institutions who had already secured funding for the project. Secondly, it could be possible that the studied diseases had a high prevalence in these African countries. For instance, Burkitt lymphoma was the most widely studied disease, and it is widespread in malaria and Epstein–Barr virus endemic regions such as Eastern Africa. Finally, the high number of participants from a specific country could be attributed to researchers affiliated with African and non-African institutions. This is one avenue through which resources could be available for a research project.

The highest level of collaboration was observed between African scientists and their American counterparts. Similar observations were made by Alali Allali, Abotsi [21] in their review on microbiome research in Africa. One attributing factor is that the American authors secured the grants and served as the principal investigators on the projects. Their labs perform all the web lab analyses, including library preparation and sequencing. Additionally, they contribute the most to data analyses and manuscript preparation. One significant role of the African collaborator has been patient recruitment and sample collection [30], which has been extensively criticized or perceived as “helicopter research” [31–33]. The funding agencies and Institutional review boards can mitigate this unfair treatment of African scientists by their foreign counterparts by including a compulsory capacity-building component in their grant calls and also for African governments should fund research in areas that disproportionally affect their populations. This will force the western researchers to equip and train their African collaborators in undertaking independent research.

## Conclusion

In this review, we have explored the RNA-seq research landscape in Africa from RNA-seq inception till February 2022. The scanty number of published studies on RNA-seq in Africa highlights the need for African governments to invest in research. Funding agencies and institutional review boards should ensure capacity-building in cutting-edge research by ensuring equitable collaboration between African and non-African researchers.

## Supplementary Information

**Additional file 1:** Contains all the search terms used in the analyses.

## Data Availability

Not applicable.
