# Supplementary material for: RNA-seq research landscape in Africa: systematic review reveals disparities and opportunities
Source: Eur J Med Res. 2023 Jul 22;28:244. doi: 10.1186/s40001-023-01206-3 (PMC10362609; doi:10.1186/s40001-023-01206-3)
Supplement: Supplementary file 1 — Additional file 1: Contains all the search terms used in the analyses. [file 40001_2023_1206_MOESM1_ESM.docx]

Supplementary Material

# Supplementary Data (Search terms)

## 1.1 PubMed

(RNA-seq OR RNA sequencing OR “RNA sequencing” OR RNA-sequencing OR mRNA sequencing OR “mRNA sequencing” OR transcriptomic OR transcriptomics OR transcriptome sequencing OR “transcriptome sequencing” OR cDNA sequencing OR “cDNA sequencing”)

AND

(Algeria OR Angola OR Benin OR Botswana OR Burkina Faso OR "Burkina Faso” OR Upper Volta OR "Upper Volta" OR Burundi OR Cameroon OR Cape Verde OR "Cape Verde" OR Central African Republic OR Chad OR Comoros OR Comoro Islands OR "Comoro Islands" OR Congo OR Democratic Republic Congo OR "Democratic Republic of the Congo" OR Zaire OR Djibouti OR Egypt OR Equatorial Guinea OR "Equatorial Guinea" OR Eritrea OR Ethiopia OR Gabon OR Gambia OR Ghana OR Gold Coast OR "Gold Coast" OR Guinea OR Guinea Bissau OR "Guinea Bissau" OR Ivory Coast OR "Ivory Coast" OR Cote d'Ivoire OR "Cote d'Ivoire" OR Kenya OR Lesotho OR Liberia OR Libya OR Libia OR Jamahiriya OR Jamahiryia OR Madagascar OR Malawi OR Mali OR Mauritania OR Mauritius OR Ile Maurice OR "Ile Maurice" OR Morocco OR Mozambique OR Moçambique OR Namibia OR Niger OR Nigeria OR Rwanda OR Sao Tome OR "Sao Tome" OR Senegal OR Seychelles OR Sierra Leone OR "Sierra Leone" OR Somalia OR South Africa OR "South Africa" OR Sudan OR South Sudan OR "South Sudan" OR Swaziland OR Tanzania OR Tanganyika OR Zanzibar OR Togo OR Tunisia OR Uganda OR Zambia OR Zimbabwe OR Northern Rhodesia OR "Northern Rhodesia" OR Southern Rhodesia OR "Southern Rhodesia" OR Africa OR Africa* OR Southern Africa OR West Africa OR Western Africa OR Eastern Africa OR East Africa OR North Africa OR Northern Africa OR Central Africa OR Sub Saharan Africa OR Subsaharan Africa OR Sub-Saharan Africa)

AND

(humans OR human OR Homo sapiens OR “Homo sapiens”)

NOT

(Single-Cell RNA Sequencing OR “Single-Cell RNA Sequencing”)

## 1.2 Scopus

(rna-seq OR "rna sequencing" OR rna-sequencing OR "mrna sequencing" OR transcriptomic OR transcriptomics OR "transcriptome sequencing" OR "cdna sequencing")

AND

( human OR humans OR "homo sapiens" )

AND NOT

( microbiome OR microbiota OR metagenomics )

AND

( LIMIT-TO ( AFFILCOUNTRY , "Algeria" ) OR LIMIT-TO ( AFFILCOUNTRY , "Angola" ) OR LIMIT-TO ( AFFILCOUNTRY , "Benin" ) OR LIMIT-TO ( AFFILCOUNTRY , "Botswana" ) OR LIMIT-TO ( AFFILCOUNTRY , "Burkina Faso" ) OR LIMIT-TO ( AFFILCOUNTRY , "Burundi" ) OR LIMIT-TO ( AFFILCOUNTRY , "Cameroon" ) OR LIMIT-TO ( AFFILCOUNTRY , "Cape Verde" ) OR LIMIT-TO ( AFFILCOUNTRY , "Central African Republic" ) OR LIMIT-TO ( AFFILCOUNTRY , "Chad" ) OR LIMIT-TO ( AFFILCOUNTRY , "Comoros" ) OR LIMIT-TO ( AFFILCOUNTRY , "republic of the Congo" ) OR LIMIT-TO ( AFFILCOUNTRY , "democratic republic of the Congo" ) OR LIMIT-TO ( AFFILCOUNTRY , "Côte D'ivoire" ) OR LIMIT-TO ( AFFILCOUNTRY , "ivory coast" ) OR LIMIT-TO ( AFFILCOUNTRY , "Djibouti" ) OR LIMIT-TO ( AFFILCOUNTRY , "Egypt" ) OR LIMIT-TO ( AFFILCOUNTRY , "equatorial guinea" ) OR LIMIT-TO ( AFFILCOUNTRY , "Eritrea" ) OR LIMIT-TO ( AFFILCOUNTRY , "Ethiopia" ) OR LIMIT-TO ( AFFILCOUNTRY , "Gabon" ) OR LIMIT-TO ( AFFILCOUNTRY , "the Gambia" ) OR LIMIT-TO ( AFFILCOUNTRY , "Ghana" ) OR LIMIT-TO ( AFFILCOUNTRY , "gold coast" ) OR LIMIT-TO ( AFFILCOUNTRY , "guinea" ) OR LIMIT-TO ( AFFILCOUNTRY , "Guinea-Bissau" ) OR LIMIT-TO ( AFFILCOUNTRY , "Kenya" ) OR LIMIT-TO ( AFFILCOUNTRY , "Lesotho" ) OR LIMIT-TO ( AFFILCOUNTRY , "Liberia" ) OR LIMIT-TO ( AFFILCOUNTRY , "Madagascar" ) OR LIMIT-TO ( AFFILCOUNTRY , "Malawi" ) OR LIMIT-TO ( AFFILCOUNTRY , "Mali" ) OR LIMIT-TO ( AFFILCOUNTRY , "Mauritania" ) OR LIMIT-TO ( AFFILCOUNTRY , "Mauritius" ) OR LIMIT-TO ( AFFILCOUNTRY , "Mayotte" ) OR LIMIT-TO ( AFFILCOUNTRY , "Morocco" ) OR LIMIT-TO ( AFFILCOUNTRY , "Mozambique" ) OR LIMIT-TO ( AFFILCOUNTRY , "Namibia" ) OR LIMIT-TO ( AFFILCOUNTRY , "Nigeria" ) OR LIMIT-TO ( AFFILCOUNTRY , "Réunion" ) OR LIMIT-TO ( AFFILCOUNTRY , "Rwanda" ) OR LIMIT-TO ( AFFILCOUNTRY , "Niger" ) OR LIMIT-TO ( AFFILCOUNTRY , "Saint Helena" ) OR LIMIT-TO ( AFFILCOUNTRY , "São Tomé and Príncipe" ) OR LIMIT-TO ( AFFILCOUNTRY , "Seychelles" ) OR LIMIT-TO ( AFFILCOUNTRY , "Sierra Leone" ) OR LIMIT-TO ( AFFILCOUNTRY , "Somalia" ) OR LIMIT-TO ( AFFILCOUNTRY , "South Africa" ) OR LIMIT-TO ( AFFILCOUNTRY , "Sudan" ) OR LIMIT-TO ( AFFILCOUNTRY , "South Sudan" ) OR LIMIT-TO ( AFFILCOUNTRY , "Swaziland" ) OR LIMIT-TO ( AFFILCOUNTRY , "Tanzania" ) OR LIMIT-TO ( AFFILCOUNTRY , "Zanzibar" ) OR LIMIT-TO ( AFFILCOUNTRY , "Togo" ) OR LIMIT-TO ( AFFILCOUNTRY , "Tunisia" ) OR LIMIT-TO ( AFFILCOUNTRY , "Uganda" ) OR LIMIT-TO ( AFFILCOUNTRY , "Zimbabwe" )

AND

( LIMIT-TO ( LANGUAGE , "English" )

AND

( LIMIT-TO ( PUBYEAR , 2022 ) OR LIMIT-TO ( PUBYEAR , 2021 ) OR LIMIT-TO ( PUBYEAR , 2020 ) OR LIMIT-TO ( PUBYEAR , 2019 ) OR LIMIT-TO ( PUBYEAR , 2018 ) OR LIMIT-TO ( PUBYEAR , 2017 ) OR LIMIT-TO ( PUBYEAR , 2016 ) OR LIMIT-TO ( PUBYEAR , 2015 ) OR LIMIT-TO ( PUBYEAR , 2014 ) OR LIMIT-TO ( PUBYEAR , 2013 ) OR LIMIT-TO ( PUBYEAR , 2012 ) OR LIMIT-TO ( PUBYEAR , 2011 ) OR LIMIT-TO ( PUBYEAR , 2010 ) OR LIMIT-TO ( PUBYEAR , 2009 ) OR LIMIT-TO ( PUBYEAR , 2008 ))

## 1.3 EBSCOhost: Academic Search Complete

(RNA-seq OR RNA sequencing OR “RNA sequencing” OR RNA-sequencing OR mRNA sequencing OR “mRNA sequencing” OR transcriptomic OR transcriptomics OR transcriptome sequencing OR “transcriptome sequencing” OR cDNA sequencing OR “cDNA sequencing”)

AND

(Algeria OR Angola OR Benin OR Botswana OR Burkina Faso OR "Burkina Faso” OR Upper Volta OR "Upper Volta" OR Burundi OR Cameroon OR Cape Verde OR "Cape Verde" OR Central African Republic OR Chad OR Comoros OR "Iles Comores" OR Iles Comores OR Comoro Islands OR "Comoro Islands" OR Congo OR Democratic Republic Congo OR "Democratic Republic of the Congo" OR Zaire OR Djibouti OR Egypt OR Equatorial Guinea OR "Equatorial Guinea" OR Eritrea OR Ethiopia OR Gabon OR Gambia OR Ghana OR Gold Coast OR "Gold Coast" OR Guinea OR Guinea Bissau OR "Guinea Bissau" OR Ivory Coast OR "Ivory Coast" OR Cote d'Ivoire OR "Cote d'Ivoire" OR Kenya OR Lesotho OR Liberia OR Libya OR Libia OR Jamahiriya OR Jamahiryia OR Madagascar OR Malawi OR Mali OR Mauritania OR Mauritius OR Ile Maurice OR "Ile Maurice" OR Morocco OR Mozambique OR Moçambique OR Namibia OR Niger OR Nigeria OR Rwanda OR Sao Tome OR "Sao Tome" OR Senegal OR Seychelles OR Sierra Leone OR "Sierra Leone" OR Somalia OR South Africa OR "South Africa" OR Sudan OR South Sudan OR "South Sudan" OR Swaziland OR Tanzania OR Tanganyika OR Zanzibar OR Togo OR Tunisia OR Uganda OR Zambia OR Zimbabwe OR Northern Rhodesia OR "Northern Rhodesia" OR Southern Rhodesia OR "Southern Rhodesia" OR Africa OR Africa* OR Southern Africa OR West Africa OR Western Africa OR Eastern Africa OR East Africa OR North Africa OR Northern Africa OR Central Africa OR Sub Saharan Africa OR Subsaharan Africa OR Sub-Saharan Africa)

AND

(humans OR human OR Homo sapiens OR “Homo sapiens”)

NOT

(Single-Cell RNA Sequencing OR “Single-Cell RNA Sequencing”)
